# Community-Acquired Cavitary *Pseudomonas* Pneumonia Linked to Use of a Home Humidifier

**DOI:** 10.1155/2017/5474916

**Published:** 2017-12-28

**Authors:** Eric Woods, Gabriel Cohen, Eric Bressman, David Lin, Nathalie E. Zeitouni, Colleen Beckford, Camille Hamula, Harm van Bakel, Mitchell Sullivan, Deena R. Altman, Daniel Caplivski

**Affiliations:** ^1^Icahn School of Medicine at Mount Sinai, New York, NY, USA; ^2^Division of Infectious Diseases, Icahn School of Medicine at Mount Sinai, New York, NY, USA; ^3^Department of Medicine, Icahn School of Medicine at Mount Sinai, New York, NY, USA; ^4^Icahn Institute for Genomics and Multiscale Biology, Icahn School of Medicine at Mount Sinai, New York, NY, USA

## Abstract

*Pseudomonas aeruginosa* is an opportunistic pathogen that rarely causes pneumonia in otherwise healthy patients. We describe a case of community-acquired *P. aeruginosa* pneumonia in a previously healthy individual who likely acquired the infection from a home humidifier.

## 1. Introduction

*Pseudomonas aeruginosa* is an opportunistic pathogen that usually causes pneumonia in patients with an underlying medical condition or a risk factor that predisposes them to infection [1]. However, several reports of severe *P. aeruginosa* pneumonia in previously healthy patients without apparent immunodeficiency have been reported [1, 2]. Only a few cases have been attributable to an identifiable source [1, 3, 4]. In this case, we describe community-acquired pneumonia (CAP) in a previously healthy 30-year-old male who we believe acquired the *P. aeruginosa* infection from a home humidifier.

## 2. Case Presentation

A 30-year-old male with no past medical history presented to the emergency department reporting three weeks of progressively worsening cough producing yellow sputum, pleuritic right scapular pain, four days of fever (to 38.9°C), and one episode of night sweats. Additionally, he noted a ten-pound weight loss, which he attributed to decreased appetite. He denied hemoptysis, shortness of breath, diarrhea, or dysuria. The patient had recently completed a five-day course of azithromycin and had been using benzonatate without relief.

In the two months prior to his presentation, he traveled through Japan, Cambodia, Thailand, Laos, and Israel. He took antimalarial medications when appropriate and had received all required travel vaccinations prior to departing. Additionally, one month before presentation, he had attended Burning Man, an artistic gathering in the middle of the Black Rock Desert in Nevada. His fiancée had traveled with him and was also experiencing a similar but less severe cough. They used a single-room humidifier nightly while sleeping. He had no history of recurrent or severe infections. He denied tobacco or alcohol use. He had a history of occasional marijuana use but denied any intravenous drug use. He worked as a teacher.

**Figure 1:**
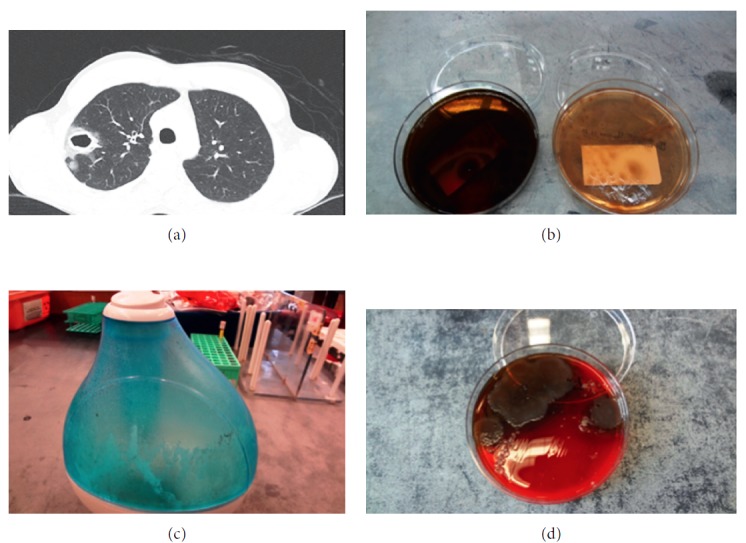
(a) Cross section of CT chest showing a right upper lobe cavitary lesion. (b) Respiratory cultures plated on blood and soy agar. (c) Humidifier with film inside water basin. (d) Humidifier culture on blood agar showing colony growth with pigment.

On physical exam, his blood pressure was 140/69 mmHg with a pulse rate of 84/min. His tympanic temperature was 36.5°C. His respiratory rate was 18/min, and oxygen saturation on room air was 99%. He was in no acute distress, and examination of his chest was clear to auscultation bilaterally. Laboratory results showed a leukocytosis (WBC 11.3 × 10^3^/*μ*L, 11.2%) without eosinophilia, an elevated venous blood lactate (3.3 mmol/L), and anemia (hemoglobin 12.9 g/dL). His initial chest x-ray was abnormal, so a CT chest with contrast was obtained, which demonstrated a 2.9 cm right upper lobe cavitary lesion (Figure 1(a)). A brief workup for immunodeficiency was then performed. The patient had a negative 4th generation HIV antigen-antibody test as well as a negative HIV RNA PCR. His CD4 count was not measured, but quantitative immunoglobulin analysis was within normal limits (IgG 875 mg/dL, IgA 186 mg/dL, and IgM 76 mg/dL). Quantiferon TB Gold was negative.

Given his clinical stability, failure of outpatient antibiotics, and inability to produce an adequate sputum sample for Gram stain, the decision was made to proceed with bronchoscopy. Bronchoscopy showed diffuse inflammation with mild thick yellow secretions. Cultures of bronchoalveolar lavage, washings, and tissue culture performed during bronchoscopy resulted with heavy growth of *P. aeruginosa* (Figure 1(b)). No other pathogens were identified. Fungal culture showed no growth after four weeks. Blood cultures, three Kinyon smears of sputum, and urine Legionella antigen were also negative. Aspergillus galactomannan antigen and (1-3)-*β*-D-glucan assay (Fungitell) were negative. Histoplasma urine antigen, serum cryptococcal antigen, and serum coccidioides antigens were not detected. *Echinococcus* antibody was not detected. Once Gram-negative bacilli were identified on cultures, he was treated with ceftazidime (1g q8h IV). The dose was increased to 2g q8h IV after speciation, and sensitivities became available. On this regimen, his condition steadily improved. He was subsequently transitioned to oral ciprofloxacin (750 mg BID for 14 days) and discharged with outpatient follow-up. He completed a 21-day antibiotic course in total.

## 3. A Search for the Source

The identification of *P. aeruginosa* as the etiologic agent of this otherwise healthy patient's chronic cough and CT chest findings prompted a review of his history for possible exposures to contaminated water. According to the patient, he had three significant exposures to aerosolized water in the past few months: nightly use of a humidifier while sleeping, being sprayed with water at Burning Man, and swimming near a waterfall in Southeast Asia. We asked the patient's family to bring his ultrasonic cool-mist humidifier from home. It was noted to have a thin gray-green film coating the plastic covering as well as standing water in the basin (Figure 1(c)). The patient was unsure when the unit had last been cleaned.

We obtained cultures of the humidifier water and basin, which both returned positive for *P. aeruginosa* (Figure 1(d)). Humidifier and patient isolates had identical patterns of antibiotic sensitivities. We performed whole genome sequencing (PacBio RSII) on the isolates obtained from the humidifier basin and from the bronchoscopy tissue. We found the isolates to be identical (accessions: NTGC01000000-NTGD01000000).

## 4. Discussion

In this report, we present the case of a young man diagnosed with cavitating *P. aeruginosa* pneumonia. By comparing antibiotic resistance patterns and using whole genome sequencing, we were able to confirm that his home humidifier was likely colonized with an identical *P. aeruginosa* isolate. The humidifier was removed from the patient's room and discarded, and he has not had any recurrence of *P. aeruginosa* pneumonia after several months of follow-up.

Our case highlights the fact that *P. aeruginosa* pneumonia in the community should prompt environmental investigations to identify possible sources of infection. The incidence of community-acquired *P. aeruginosa* pneumonia in otherwise healthy individuals is low. In one study, the *P. aeruginosa* pathogen was isolated in 0.4% of patients diagnosed with CAP [5]. A previous review of published cases of *P. aeruginosa* CAP in previously healthy adults using strict criteria showed only 12 cases [2]. As noted, isolated cases have been reported in which environmental sources including a home-humidifying device and a whirlpool spa have been identified.

We suspect that the patient described in this report acquired *P. aeruginosa* from the contaminated water in his humidifier. Maintenance of this patient's humidifier was inadequate; stagnation of tap water without sterilization of the machine likely permitted production of an increased bacterial load and biofilms. Home humidifiers, particularly cool-mist and ultrasonic units, like the one possessed by this patient, have been shown to aerosolize their contents [6]. When the fluid in the reservoir of a humidifier becomes contaminated with bacteria, the aerosol may contain high concentrations of bacteria [7]. To obtain a broader perspective on the microbiology of single-room humidifiers, Hull et al. sequenced rRNA obtained from 26 home humidifiers [8]. The authors found that bacteria encountered were generally low in diversity and were dominated by the orders *Sphingomonadales*, *Rhizobiales*, and *Burkholderiales*. These are all types of bacteria that can form biofilms and are commonly found in environmental water sources. Very few sequences of *P. aeruginosa* were found [8]. However, another study of respiratory devices in multiple medical settings found a surprisingly high (21% of 70 samples) rate of *P. aeruginosa* contamination [9].

Patients such as ours reinforce the possibility for a rare but potentially life-threatening adverse effect of home humidifier use. Similar to the patient in this case, many other individuals fail to adequately clean their home humidifiers. According to a survey conducted by Consumer Reports, 40% of Americans rarely or never clean their humidifiers [10]. In accordance with the Centers for Disease Control and Prevention guidelines for the prevention of nosocomial pneumonia, room-air humidifiers that create aerosols should not be used unless they can be sterilized or subjected to daily disinfection and filled only with sterile water [11]. In addition to frequent disinfection of the device to avoid microbial colonization, performing periodic surveillance of the humidifier components to look for microbial growth would also be prudent. Finally, as whole genome sequencing is becoming a more readily available technology in the clinical space, it proved useful in confirming that the patient and environment isolates were identical [12].
