# Corrigendum to “Community-Acquired Cavitary *Pseudomonas* Pneumonia Linked to Use of a Home Humidifier”

**DOI:** 10.1155/2018/6548482

**Published:** 2018-06-05

**Authors:** Eric C. Woods, Gabriel M. Cohen, Eric Bressman, David Lin, Nathalie E. Zeitouni, Colleen Beckford, Camille Hamula, Harm van Bakel, Mitchell Sullivan, Deena R. Altman, Daniel Caplivski

**Affiliations:** ^1^Icahn School of Medicine at Mount Sinai, New York, NY, USA; ^2^Division of Infectious Diseases, Icahn School of Medicine at Mount Sinai, New York, NY, USA; ^3^Department of Medicine, Icahn School of Medicine at Mount Sinai, New York, NY, USA; ^4^Icahn Institute for Genomics and Multiscale Biology, Icahn School of Medicine at Mount Sinai, New York, NY, USA

In the article titled “Community-Acquired Cavitary *Pseudomonas* Pneumonia Linked to Use of a Home Humidifier” [1], the names of the first and the second authors were given incorrectly as Eric Woods and Gabriel Cohen. The authors' names should have been written as Eric C. Woods and Gabriel M. Cohen. The revised author list is shown above.
